# Differences in the Nemosis Response of Normal and Cancer-Associated Fibroblasts from Patients with Oral Squamous Cell Carcinoma

**DOI:** 10.1371/journal.pone.0006879

**Published:** 2009-09-01

**Authors:** Kati Räsänen, Ismo Virtanen, Pertteli Salmenperä, Reidar Grenman, Antti Vaheri

**Affiliations:** 1 Haartman Institute, University of Helsinki, Helsinki, Finland; 2 Institute of Biomedicine/Anatomy, University of Helsinki, Helsinki, Finland; 3 Department of Otorhinolaryngology - Head and Neck Surgery, Turku University Central Hospital, Turku, Finland; New Mexico State University, United States of America

## Abstract

**Background:**

Tumor-stroma reaction is associated with activation of fibroblasts. Nemosis is a novel type of fibroblast activation. It leads to an increased production of growth factors and proinflammatory and proteolytic proteins, while at the same time cytoskeletal proteins are degraded. Here we used paired normal skin fibroblasts and cancer-associated fibroblasts (CAF) and primary and recurrent oral squamous cell carcinoma (SCC) cells to study the nemosis response.

**Principal Findings:**

Fibroblast nemosis was analyzed by protein and gene expression and the paracrine regulation with colony formation assay. One of the normal fibroblast strains, FB-43, upregulated COX-2 in nemosis, but FB-74 cells did not. In contrast, CAF-74 spheroids expressed COX-2 but CAF-43 cells did not. Alpha-SMA protein was expressed in both CAF strains and in FB-74 cells, but not in FB-43 fibroblasts. Its mRNA levels were downregulated in nemosis, but the CAFs started to regain the expression. FSP1 mRNA was downregulated in normal fibroblasts and CAF-74 cells, but not in CAF-43 fibroblasts. Serine protease FAP was upregulated in all fibroblasts, more so in nemotic CAFs. VEGF, HGF/SF and FGF7 mRNA levels were upregulated to variable degree in nemosis. CAFs increased the colony formation of primary tumor cell lines UT-SCC-43A and UT-SCC-74A, but normal fibroblasts inhibited the anchorage-independent growth of recurrent UT-SCC-43B and UT-SCC-74B cells.

**Conclusions:**

Nemosis response, as observed by COX-2 and growth factor induction, and expression of CAF markers α-SMA, FSP1 and FAP, varies between fibroblast populations. The expression of CAF markers differs between normal fibroblasts and CAFs in nemosis. These results emphasize the heterogeneity of fibroblasts and the evolving tumor-promoting properties of CAFs.

## Introduction

Tumor microenvironment plays a major role in cancer progression and fibroblasts are known to be key components of the tumor stroma. Recently it has been suggested that stromal fibroblasts initially inhibit early stages of carcinogenesis and later under the paracrine influence of the transformed epithelia become activated leading to promotion of cancer growth. The dependence of carcinomas on stromal fibroblasts decreases as the cancer progresses, partly through a switch in epithelial cells from paracrine to autocrine regulation [1], [2]. Among the activated fibroblasts are cancer-associated fibroblasts (CAF), that are characterized by increased mitotic index, mutations in tumor suppressor genes such as p53 and by increased secretion of growth factors, chemokines and components of extracellular matrix (ECM) [2], [3], changes which all are involved in invasion and tumor growth [4].Widely used CAF markers include α-smooth muscle actin (α-SMA), fibroblast specific protein 1 (FSP1, also known as S100A4) and fibroblast activation protein (FAP, also known as seprase) [5]. α-SMA, a component of the cytoskeleton, is the most often used marker for activated fibroblasts. It becomes incorporated into stress fibers thereby augmenting the contractile activity of the fibroblasts [6]. FSP1 belongs to the S100 super-family of calcium-binding proteins. It promotes tumor growth by regulating cell cycle progression and cytoskeletal integrity [7]. FAP is a serine protease that is not expressed in normal adult tissues, but its expression is induced in activated fibroblasts responding to wound healing and tumor-stroma reaction [8]. However, it is well established that fibroblasts are heterogeneous [9], [10] and that CAFs differently express these markers [11], [12].

Nemosis, a phenomenon of fibroblast activation (for review see Vaheri et al. 2009 [13]), has previously been studied using normal dermal fibroblasts [14]–[19]. Formation of a fibroblast spheroid causes myriad of genes to be differentially expressed in these activated fibroblasts. Two distinct patterns can be found in the expression: i) expression of growth factors and proteolytic and proinflammatory proteins increases and ii) expression of cytoskeletal components decreases. Based on previously published results, cyclooxygenase-2 (COX-2), that is known to be associated with inflammation and early stages of carcinogenesis, and hepatocyte growth factor / scatter factor (HGF/SF), which has been shown to promote tumor cell invasiveness, have been considered hallmark proteins of nemosis. Spontaneous clustering of fibroblasts into spheroids can also be induced by tumor cell conditioned medium [14], [15]. We have previously shown that culturing fibroblast spheroids under the influence of benign HaCaT keratinocytes inhibits nemosis, as seen by suppressed expression of COX-2, whereas malignant HaCaT cells have a nemosis-promoting effect on normal fibroblasts, manifested as enhanced upregulation on COX-2, HGF/SF and VEGF (vascular endothelial growth factor) [17].

Head and neck squamous cell carcinoma (HNSCC) is the sixth most common malignancy worldwide and the overall patient survival is poor. This is mainly due to high rates of cancer recurrence and local invasion, partly caused by p53 gene mutations, which can be found in more than 70% of HNSCCs [20], [21]. Surgery and radiotherapy are the most commonly used lines of treatment and currently the only approved molecular targeted therapy for head and neck cancer is cetuximab (Erbitux; ImClone Systems Inc., New York, NY), a monoclonal antibody inhibitor of epidermal growth factor receptor (EGFR). However, not all HNSCC patients benefit from EGFR-targeted therapies, since overexpression, but not mutation seems to determine the treatment response. Phase 3 trials for HNSCC are currently underway for targeting VEGF (Bevacizumab, monoclonal antibody inhibitor) and for p53 (INGN 201, gene therapy) [22], [23]. Another potential target is COX-2 that has been found to be elevated in oral squamous cell carcinoma (OSCC) and has been shown to decrease tumor radiosensitivity [24]. Studies using matched patient cell strains have showed that there is a correlation in radiosensitivities between OSCC cells, dermal fibroblasts and cancer-associated fibroblasts collected from the same individual and that these individual differences in the radiosensitivity might predict the outcome of radiotherapy [25], [26].

Based on the previous results that under the influence of malignant cells normal nemotic fibroblasts start to resemble CAFs, the objective of this work was to study the nemosis response of autologous skin and cancer-associated fibroblasts, to compare the expression of CAF markers between these fibroblasts strains and their fate in nemosis and to investigate how these different fibroblast populations influence the patient-matched oral SCC cells. Our study shows that both normal and cancer-associated fibroblasts show variation between individuals, seen as varying basal CAF expression levels and different growth factor responses in nemosis, and have a differential impact on the SCC cells. The behaviour of the studied CAF markers in nemosis followed the general nemosis response: cytoskeletal α-SMA and FSP1 were downregulated and proteolytic FAP was upregulated. The only exception was one of the CAF strains that upregulated FSP1 in nemosis. Major systematic differences between normal and cancer-associated fibroblasts were the decreased basal levels of growth factors in CAFs and the capability of nemotic CAFs to start to regain the α-SMA expression and the increased FAP expression in nemosis compared to their normal counterparts.

## Results

### Different fibroblast populations show differences in response to nemosis

**Figure 1:**
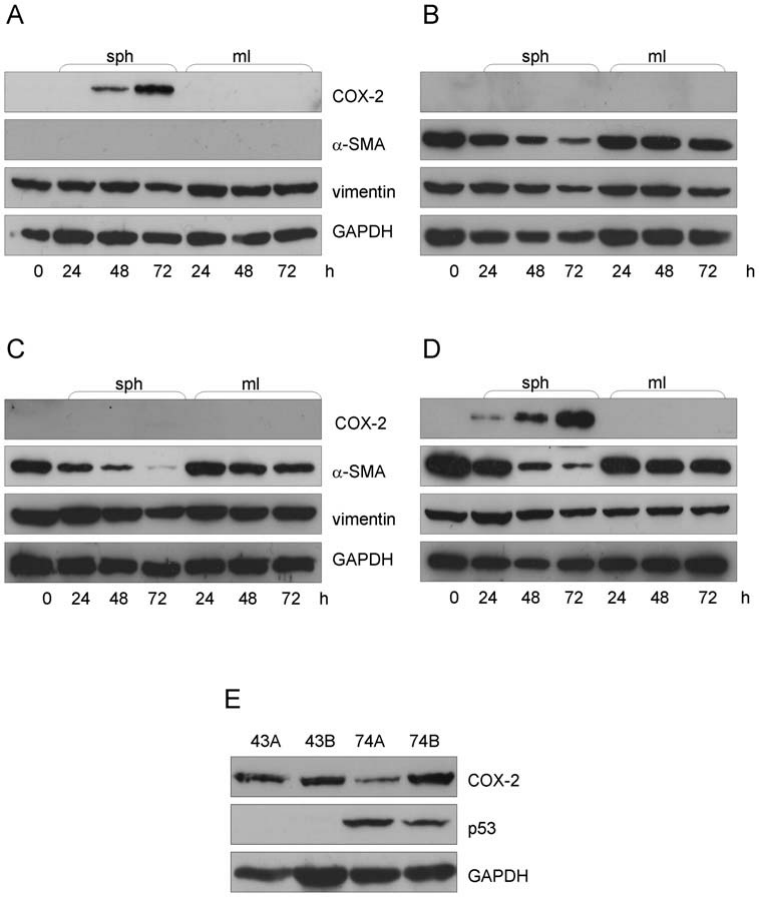
Nemosis response in different fibroblast populations. Fibroblasts were grown as spheroids or monolayer for the time indicated. (A) FB-43 spheroids started to produce COX-2 after 48 hours and no α-SMA was produced, whereas CAF-43 cells (B) did not induce COX-2 but expressed α-SMA. Both FB-74 (C) and CAF-74 (D) produced α-SMA, but COX-2 was only induced in CAF-74 spheroids. All fibroblasts types expressed equal amounts of vimentin. (E) All UT-SCC cells expressed COX-2, but only 74A and 74B showed induced p53 levels.

First we wanted to investigate the expression of the previously used nemosis marker COX-2 in the four fibroblast populations. There was no basal expression of COX-2 in any of the fibroblast strains, and it was not induced in monolayer culture. However, when cultured as spheroids the normal fibroblasts FB-43 started to express COX-2 after 48 hours (Figure 1A), but this was not seen in the other normal fibroblasts strain FB-74 (Figure 1C). Opposite results were seen with the cancer-associated fibroblasts, where no COX-2 was expressed in the CAF-43 fibroblast spheroids (Figure 1B) but the CAF-74 cells started to express COX-2 after 24 hours (Figure 1D). These results differ from previously published and indicate that COX-2 should not be solely used to measure nemosis response.

We also looked at the protein levels of vimentin and α-SMA in these cells. All four fibroblast populations expressed vimentin in equal amounts, as expected, since fibroblasts *in vitro* are considered to be in a state resembling wound healing. Both CAF strains expressed α-SMA, CAF-74 slightly more than CAF-43. Interestingly, also the normal FB-74 cells expressed α-SMA, but no protein expression was detected in the other normal fibroblast cell strain FB-43. Time-dependent downregulation of α-SMA was seen in spheroids but not in the monolayer cultures, caused by the degradation of cytoskeleton in these fibroblasts going through nemosis. This is in line with previous results by Bizik et al. [14], where decreasing actin levels were used as a marker of spheroid degradation. GAPDH was used as a loading control.

Figure 1E is an immunoblot of the four UT-SCC carcinoma cell lines. All of them expressed COX-2, but interestingly only UT-SCC 74A and 74B had an induced p53 protein level, suggesting a p53 mutation. We could not detect p53 in any of the fibroblast populations, a notion that concurs with the report by Qiu et al. [27] in which they could not detect somatic genetic alterations in CAFs.

### Different expression of CAF markers in fibroblast strains

**Figure 2:**
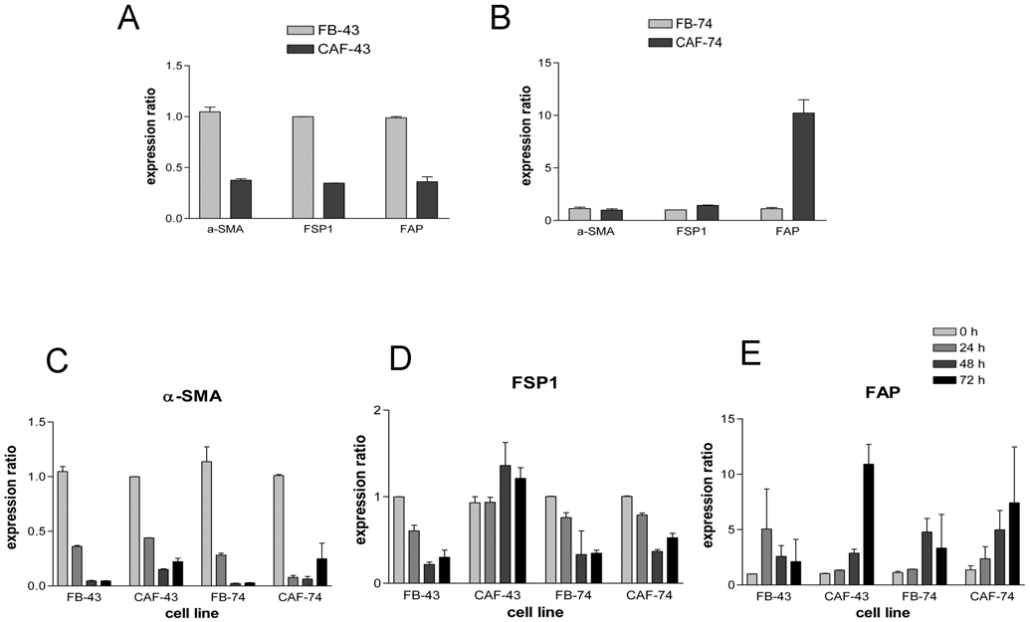
CAF marker mRNA expression. Gene expression of CAF markers was studied using Q-PCR. (A) α-SMA, FSP1 and FAP expression ratios were significantly lower (P<0.01) in CAF-43 cells compared to FB-43 cells, but equal or higher in CAF-74 fibroblasts. (B) When grown as spheroids all fibroblasts downregulated α-SMA expression, but CAFs started to regain the expression at 72 h (P<0.05 in FB-43 vs. CAF-43 and in FB-74 vs. CAF-74) (C) FSP1 was downregulated in normal fibroblasts and in CAF-74 cells, but not in CAF-43 cells, (D) and FAP was upregulated in all cell lines going through nemosis, more so in CAFs (P<0.05 in 72 h CAF-43 when compared to 72 h FB-43 ). Columns: mean; error bars; SEM.

Since the protein levels of α-SMA varied between different fibroblast populations, we decided to investigate also the expression of other widely used CAF markers FSP1 and FAP. Gene expression pattern of these three genes in the fibroblasts grown as spheroids for 0, 24, 48 and 72 hours was analyzed using quantitative real-time PCR. Q-PCR was chosen as the method over immunoblotting because of its higher sensitivity. GAPDH was used as a reference gene that the expression of target genes was normalized to, after which relative fold expression ratios were calculated. The basal expression level of α-SMA, FSP1 and FAP was significantly lower (P<0.01) in CAF-43 cells than in normal FB-43 fibroblasts (Figure 2A). However, as seen in Figure 2B, this was not the case with CAF-74 fibroblasts, where compared to FB-74 cells α-SMA expression ratio was equal, FSP1 1.4-fold higher and FAP expression ratio surprisingly 10-fold higher (P<0.01).

Nemosis response of the CAF markers between these fibroblast populations showed also variation. The α-SMA level was drastically downregulated in spheroids, reflecting the protein levels and indicating the decomposition of cytoskeleton in these spheroids. This was also true for FB-43 cells, for which we could not detect protein expression. Differing from the normal fibroblasts, the CAFs started to regain the α-SMA expression at 72 hours; when compared to normal fibroblasts the increase was statistically significant (P<0.05) (Figure 2C). FSP1 mRNA decreased, as expected, in both normal fibroblast cell strains and in CAF-74 cells, but surprisingly increased in CAF-43 spheroids (Figure 2D). The third CAF marker FAP was induced in nemosis in all fibroblast populations, following the general nemosis fingerprint. This induction was higher in CAF spheroids than in normal fibroblast spheroids (P<0.05 in CAF-43 vs. FB-43, not statistically significant in 73 cells due to high variation between samples) (Figure 2E).

### Differential expression of growth factors in normal and cancer-associated fibroblasts

**Figure 3:**
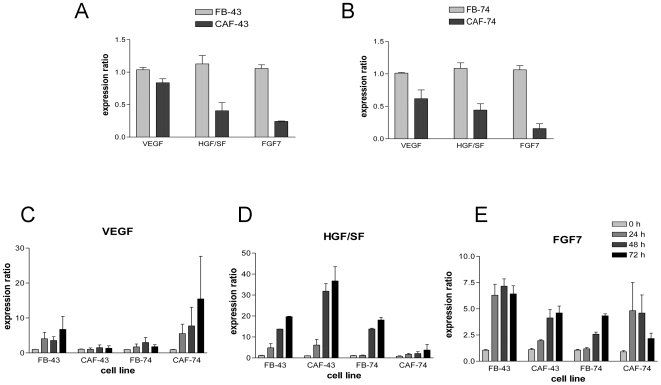
Growth factor mRNA levels. Growth factor gene expression was studied using Q-PCR. (A and B) Both CAF cell lines had reduced expression of HGF/SF (P<0.05) and FGF7 (P<0.01) and all three growth factors were upregulated to a varying degree in nemosis (C – VEGF, D – HGF/SF and E – FGF7). Columns: mean; error bars; SEM.

The other hallmark of nemosis is the increased production of several growth factors, including VEGF, HGF/SF and FGF7 (KGF). Therefore we used Q-PCR to study the expression levels of these genes in the four fibroblast populations. Both CAF strains had lower basal expression levels of VEGF, HGF/SF (P<0.05) and FGF7 (P<0.01) mRNA compared to the paired normal fibroblasts (Figure 3A and 3B). When grown as spheroids all four fibroblast populations showed upregulated VEGF, HGF/SF and FGF7 mRNA expression. VEGF induction was highest in CAF-74 cells (over 15-fold) (Figure 3C), whereas highest HGF/SF induction was seen in CAF-43 cells (over 30-fold) (Figure 3D). FGF7 levels were regulated slightly differently; over 6-fold induction was observed in FB-43 cells, other cells had an average of 5-fold induction, and interestingly in CAF-74 cells at the 72 hour time point this induction had come down to 2.5-fold (Figure 3E).

### Paracrine regulation between fibroblasts and SCC cells

**Figure 4:**
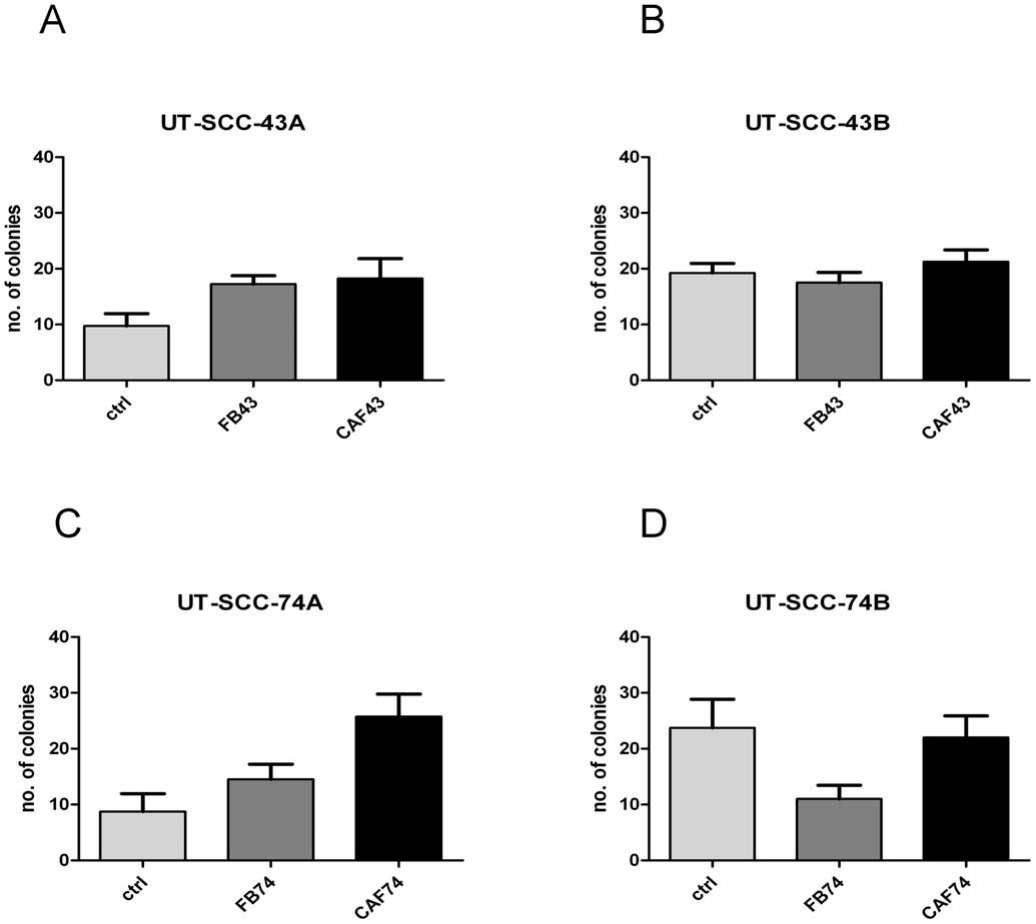
Soft-agarose assay scores. UT-SCC colony formation was studied with soft-agarose assay. All UT-SCC cells formed colonies in soft agarose, recurrent SCC (B and D) twice as many as primary SCC cells (A and C) (P<0.05). Normal fibroblasts increased the number of colonies of primary carcinomas cells and this was further augmented with CAF cells (P<0.05) (A and C). Recurrent SCC cell colony formation was inhibit with normal fibroblasts (P<0.05 in FB-74 compared to control) and restored to control level by CAFs (B and C). Columns: mean; error bars; SEM.

Anchorage-independent growth of UT-SCC carcinoma cell lines was tested using the soft-agarose assay. During the three-week observation period all four carcinoma cell lines formed colonies, but clear difference between primary and recurrent tumor cell lines was seen (Figure 4). When cultured alone, both recurrent tumor cell lines UT-SCC-43B and UT-SCC-74B formed twice the amount of colonies compared to primary tumor cell lines UT-SCC-43A and UT-SCC-74A; the difference was statistically significant in both cases (P<0.05). The underlying monolayer of normal fibroblasts FB-43 and FB-74 increased slightly the number of 43A and 74A carcinoma cell colonies, respectively. Increase in colony numbers was further augmented when 43A and 74A SCC cells were grown under the influence of CAF-43 and CAF-74 (P<0.05), respectively. Interestingly, when culturing the more invasive 43B and 74B SCC cells with FB-43 and FB-74 fibroblasts, a decrease in colony number was seen; this effect was more pronounced with 74B cells (P<0.05 in FB-74 compared to control). CAF-43 and CAF-74 fibroblasts restored the number of colonies to same level as control, but did not further enhance the colony formation of recurrent SCC cells.

**Figure 5:**
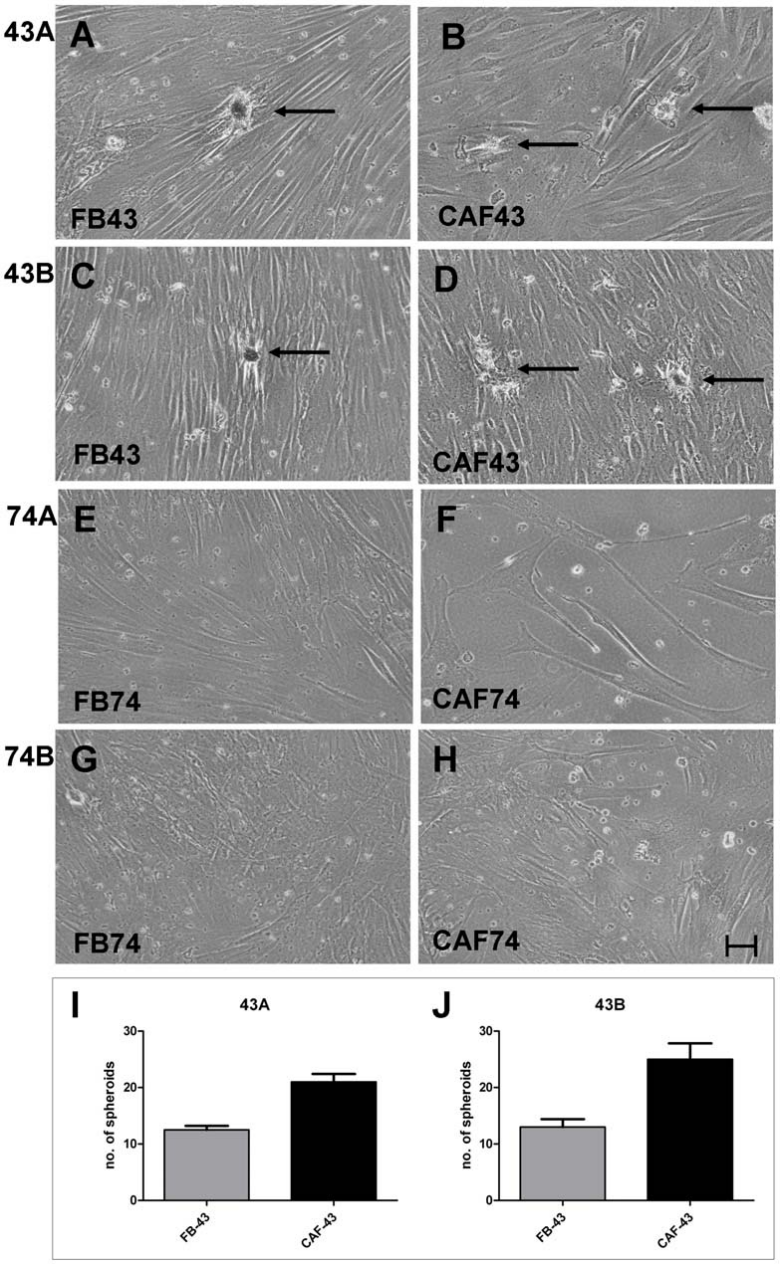
Fibroblast growth in soft-agarose assay. Representative pictures of underlying fibroblast monolayer cultures. Spontaneous clustering (arrows) is seen in FB-43 and CAF-43 cells under the influence of paired SCC cells 43A (A and B) and 43B (C and D). In contrast, FB-74 (E and G) and CAF-74 (F and H) did not form spheroids. Scale bar 60 µm. The number of formed spheroids was calculated from the monolayer fibroblast cultures (I and J). CAF-43 cells formed significantly more spheroids than FB-43 cells (P<0.05). Columns: mean; error bars; SEM.

The UT-SCC colony formation results were in line between cells obtained from the two individuals; however, there was variation between individuals when observing closely the underlying monolayer fibroblast cultures. Spontaneous spheroid formation was seen in the underlying monolayer culture of FB-43 and CAF-43 fibroblasts when co-cultured with both 43A and 43B SCC cells (Figure 5A–D). FB-74 and CAF-74 did not spontaneously form spheroids under any of the above conditions, but they did grow faster when co-cultured with more malignant 74B cells (Figure 5G–H). Figures 5I–J presents the spontaneous spheroid formation of 43 fibroblasts with 43A and 43B SCC cells. With both UT-SCC cell lines, CAF-43 cells formed significantly more spheroids than FB-43 fibroblasts (P<0.05). The two different SCC cell lines did not influence significantly the fibroblast spheroid formation; although a slight increase was seen in CAF-43 spheroids with recurrent 43B SCC cells. None of the fibroblast types (FB-43, CAF-43, FB-74 and CAF-74) formed colonies in soft agarose.

**Figure 6:**
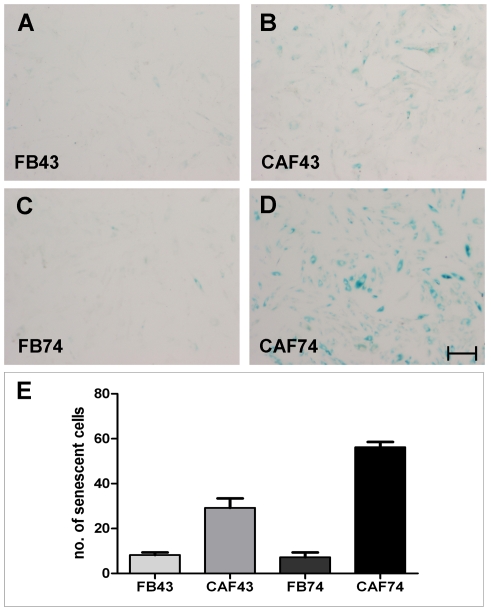
Senescence-associated beta-galactosidase activity of fibroblasts. Representative pictures of fibroblast monolayer cultures stained for SA-β-gal activity. FB-43 (A) and FB-74 (C) show little or no staining; CAFs are positive (B and D). Scale bar 200 µm. CAF-74 had significantly more (P<0.01) SA-β-gal cells compared to CAF-43 (E). Columns: mean; error bars; SEM.

To elucidate the reason for the different behavior of the fibroblast strains on monolayer cultures, and particularly the slow growth rate of CAF-74 cells, we performed senescence-associated beta-galactosidase (SA-β-gal) staining. SA-β-gal activity is the most commonly used marker for cellular senescence. Premature stress-induced senescence is caused by oxidative stress, DNA damage and oncogene activation [28]. As expected, CAF-74 cells showed strong SA-β-gal staining. CAF-43 fibroblasts were as well positive, but CAF-74 had significantly more (P<0.01) senescent cells (Figure 6).

## Discussion

Primary carcinomas are considered to be unorganized organs that are composed of various cell types, including cancer cells, fibroblasts and other mesenchymal cells, and cells related to immunity and vasculature. The tumor-stroma microenvironment leads to fibroblast activation and paracrine signaling between fibroblasts and cancer cells [29]. In nemosis, activated fibroblasts start to produce proteins involved in inflammation, proteolysis and cancer progression and at the same time downregulate the expression of cytoskeletal proteins [14]–[19].

The objective of this work was to investigate the nemosis response of patient-matched normal and cancer-associated fibroblasts, and to study the expression pattern of CAF markers and their behaviour in nemosis. Only one of the normal fibroblast strains (FB-43) and one of the CAFs (FB-74) induced COX-2 in nemosis. This is in contrast with previously published results, where COX-2 induction has been considered a hallmark feature of nemosis. However, in those studies fibroblasts have been from neonatal origin and here we have used fibroblasts obtained from adults. This conflicting result therefore indicates that COX-2 should not be solely used to measure nemosis response, but other markers, such as the profile of secreted proteins, should be investigated as well.

The other key feature of nemosis is the time-dependent degradation of cytoskeleton. On protein level three of the fibroblast strains expressed α-SMA, surprisingly also the normal skin fibroblasts FB-74. The other normal fibroblast strain FB-43 did not express α-SMA at the protein level. However, when measuring the mRNA levels with the more sensitive Q-PCR method, all four fibroblast populations showed α-SMA expression and this was downregulated in nemosis. The time-dependent downregulation on both protein and mRNA level is in line with previous results on nemosis, indicating the decomposition of the cytoskeleton. Interestingly the CAFs started to regain the α-SMA mRNA expression at 72 h, the difference was significant when compared to their normal counterparts. In contrast to our results, a study by Shannon et al. [30] showed that normal skin fibroblasts, but not oral fibroblasts expressed α-SMA. However, more recent results showed, in line with our results, that normal oral fibroblasts express α-SMA and this expression increased when these cells were cultured in conditioned medium obtained from OSCC cells [31]. These contradicting results might come partly from the method that was used to measure the α-SMA expression; in the first one immunoblotting was used, in the second the method was slightly more sensitive immunohistochemistry.

We investigated also the mRNA levels of two other CAF markers, FSP1 and FAP. In nemosis FSP1 levels decreased in FB-43, FB-74 and CAF-74 spheroids, but increased in CAF-43 cells. The third investigated CAF marker FAP was upregulated in nemosis, more in CAFs than in normal fibroblasts, the difference was significant with the 43 fibroblast strains. With all three CAF markers the nemosis response followed the pattern of decreased expression of cytoskeletal genes (α-SMA and FSP1) and increase in proteolytic gene expression (FAP). Clearly different response was seen with CAF-43 cells where, instead of downregulation of FSP1, the levels increased in nemosis.The heterogeneity of fibroblasts becomes evident when looking at the basal levels of the CAF marker expression; CAF-43 cells had lower levels of all three markers, CAF-74 had less α-SMA, slightly more FSP1 and over 10-fold more FAP. These results also emphasize that α-SMA, the most commonly used CAF / myofibroblast marker, should not be used solely to define activated fibroblasts.

Another hallmark of nemosis is the induction of growth factors. It has been shown that oral fibroblasts produce significantly more FGF7 and HGF/SF when compared to skin fibroblasts [30]. These two growth factors, together with VEGF, are known to be important in wound repair and cancer progression [32], [33]. The basal expression of VEGF, HGF/SF and FGF7 mRNA was lower in CAFs than in normal fibroblasts, and this is in contrast to previous results [30]. However, the growth rate of these cells was slower than that of their normal counterparts, which might reflect their decreased production of growth factors. The SA-β-gal activity of the CAFs supports this theory, indicating that these cells are senescent. The need for these growth factors to be secreted by fibroblasts could be reduced in the CAFs since the tumor cells themselves, along with infiltrated macrophages and endothelial cells, are capable to produce these factors. As expected, VEGF, HGF/SF and FGF7 mRNAs were upregulated in fibroblast nemosis, and the level of induction varied between fibroblast populations. VEGF induction was highest in CAF-74 spheroids, HGF/SF in CAF-43 spheroids and FGF7 in FB-43 spheroids.

Based on these results it seems that the capability of normal and cancer-associated fibroblasts to produce these growth factors in nemosis is somewhat related to the extent they are needed in cancer progression. The dependence of tumors on stromal fibroblasts, and particularly on the growth factors they produce, decreases in the course of tumor progression. Epithelial cells require FGF7 to break the epithelial polarization. FGF7 is only expressed by stromal cells and its receptor FGFR2IIb only by epithelial cells, indicating the role FGF7 in the beginning of tumor progression [34]. Of the studied fibroblast populations the FB-43 cells, which appear to be most normal of the studied strains (based of induction of COX-2 and lack of α-SMA), had the highest FGF7 induction in nemosis. HGF/SF is required for the migration / scattering of the epithelial cells from the initial break point. Nemotic CAF-43 cells produced more HGF/SF than the other three cell strains. Supporting this Kankuri et al. [15] have shown that HGF/SF produced by fibroblast spheroids directly promotes cancer cell invasion. Also another study has shown that oral fibroblasts drive invasion of OSCC cells by increasing secretion of HGF/SF [35]. VEGF is required later in the tumor progression when the cancer cell mass extends the point where it can no longer grow without oxygen supply. VEGF, secreted by fibroblasts, induces angiogenesis by recruiting endothelial cells to form new blood vessels [36]. CAF-74 cells, which are senescent, have by far the highest level of VEGF in nemosis.

It has been well established that CAFs, but not normal fibroblasts, are capable to promote tumor progression [37]–[39]. More recent results have shown that initially the normal fibroblasts inhibit the growth of cancer cells [2], and our present results concur with that notion. We show here that normal fibroblasts indeed inhibit the colony formation of recurrent SCC cells, but curiously this was not seen with primary tumor cells. The CAFs seem to be able to influence only the primary SCC cells and not the recurrent cells. The CAFs produced lower levels of growth factors, and it could be that for this reason they are capable to influence the more responsive primary SCCs, but the less sensitive recurrent cells do not respond to this lower amount of secreted growth factors.

The observed spontaneous spheroid formation of FB-43 and CAF-43 in monolayer cultures is in line with the results from Kankuri et al. [15], where spontaneous clustering of fibroblasts, i.e. nemosis, could be achieved by adding tumor cell-derived conditioned medium to a fibroblast monolayer. However, we did not find this with the fibroblast strains from the other SCC patient. This might be partly due to the induction of tumor suppressor p53 in the 74A and 74B SCC cells. Nonetheless, the fibroblasts did grow faster under the influence of 74B SCC cells; this was also true with the CAF-74 cells that seem to be in a state of stress-induced senescence. Further more, we did not see anchorage-independent growth of the fibroblasts, conflicting with the results obtained with prostate- and prostate carcinoma associated fibroblasts [40]. Possible explanations for this are individual variations and the origin of the fibroblasts. It is worth noting that in the soft-agarose experiments the SCC cells and fibroblasts were not in direct contact but separated by a solid layer of agarose, and the cultures were not replenished by fresh medium. Therefore the paracrine signaling between these two cell types must be mediated by soluble factors.

In conclusion, this study clearly demonstrates that fibroblasts obtained from different individuals vary in gene expression and behavior and that the expression of CAF markers differs between normal fibroblasts and CAFs in nemosis. Both normal and cancer-associated fibroblasts modulate tumor cells, normal fibroblasts by inhibiting the growth of invasive SCC cells and CAFs by further enhancing the growth of primary SCC cells. Nemosis, an *in vitro* model of fibroblast activation, may have its *in vivo* counterpart in cancer-associated fibroblasts and is a valuable tool in studying the variations between fibroblasts obtained from different individuals. Nemosis response, particularly of the CAF markers α-SMA and FAP, could therefore be used as a prognostic marker to predict the stromal reaction of tumors.

## Materials and Methods

### Cell strains and cell culture

All used cell strains had been previously established [25], [26], [41] and were provided by Dr Reidar Grenman (Turku University Central Hospital, Finland). In brief, UT-SCC-43A (43A) cells were obtained from primary tumor of a 75-year old female with gingival ulceration and metastasis. Histology (T4N1M0) was a well-differentiated SCC. UT-SCC-43B (43B) cells were established from the resected recurrent tumor. UT-SCC-74A (74A) cells were obtained from a 31-year old male having SCC in lingual right margin (T3N1M0). UT-SCC-74B (74B) cell line was established from a metastasis found later in the neck. The patient-matched FB-43 and FB-74 normal fibroblasts were obtained from the skin and CAF-43 and CAF-74 fibroblasts were obtained from the stroma of the respective oral SCC. The mesenchymal origin of fibroblast strains was originally confirmed by positive staining for vimentin and negative staining for cytokeratin using immunohistochemistry.

All cell populations were cultured at +37°C in 5% CO_2_ atmosphere in Dulbecco's modified Eagle's medium (DMEM) (Invitrogen, Carlsbad, CA) and supplemented with 5% fetal calf serum (FCS) (Invitrogen), 0.3 mg/ml glutamine, 100 µg/ml streptomycin and 100 U/ml penicillin. Fibroblast spheroids were formed as described previously [17]. In brief, 150-µl aliquots/well of single cell suspensions (1.3×10^5^ cells/ml) were plated on agarose-coated U-bottom 96-well plates (Costar, Cambridge, MA). Monolayer cultures were plated at the same density either on flat-bottomed 96-well plates (for immunoblotting) or on 6-cm dishes (for Q-PCR) (Greiner Bio-One, Frickenhausen, Germany). Cells were harvested at 24 h, 48 h and 72 h. As zero-hour time point the single cell suspension at the time of seeding was used. Fibroblasts were used till passage number 20 and UT-SCC cell lines till passage number 55.

### Immunoblotting

The samples were harvested in 2× sample buffer (125 mM Tris (pH 6.8), 4% sodium dodecyl sulfate (SDS), 0.01% bromophenol blue, 10% β-mercaptoethanol, 10% glycerol) and equal amounts of protein from each sample were resolved by 10% SDS-PAGE. Proteins were transferred to nitrocellulose membrane (Schleicher & Schuell, Dassel, Germany) and blocked with 2.5% non-fat powdered milk in TBS (20 mM Tris-HCl pH 7.5, 150 mM NaCl and 0.1% Tween-20).

The following primary antibodies were used: rabbit polyclonal anti-COX-2 (Labvision, Fremont, CA), rabbit polyclonal anti-GAPDH (Santa Cruz Biotechnology, Santa Cruz, CA), mouse monoclonal anti-α-SMA (DakoCytomation, Glostrup, Denmark), mouse monoclonal anti-vimentin (65EE3; [42]) and mouse monoclonal anti-p53 (DO-1, Thermo Fisher Scientific, Cheshire, UK ) and secondary antibodies: horseradish peroxidase coupled anti-rabbit IgG (Santa Cruz) and anti-mouse IgG+IgM (Jackson Immunoresearch, Cambridgeshire, UK). Immunoreactive proteins were visualized using ECL detection (Pierce, Rockford, IL).

### Real-time quantitative PCR

The samples for Q-PCR were harvested in RNAprotect Cell Reagent and total RNA was extracted according to the manufacturer's instructions using RNeasy Plus Mini kit (Qiagen, Hilden, Germany). Using the SuperScript VILO cDNA synthesis kit (Invitrogen) 500 ng of RNA from each sample were reverse-transcribed according to the manufacturer's instructions. Real-time quantitative PCR was done using DyNAmo Capillary SYBR Green Quantitative PCR kit (Finnzymes, Espoo, Finland) with a LightCycler Instrument (Roche Applied Science, Mannheim, Germany). Primer sequences are listed in Table 1; primers were purchased from Oligomer (Helsinki, Finland). Relative expression of target gene mRNA referenced to GAPDH was calculated using the REST-MSC software's Pair-Wise Fixed Reallocation Randomization Test [43], [44].

**Table 1:** Primer sequences used in Q-PCR.

Gene	Forward primer 5′–3′	Reverse primer 5′– 3′
GAPDH	ATGGGGAAGGTGAAGGTCG	TAAAAGCAGCCCTGGTGACC
VEGF	TACCTCCACCATGCCAAGTG	ATGATTCTGCCCTCCTCCTTC
HGF/SF	CGCTGGGAGTACTGTGCAAT	CCCTGTAGCCTTCTCCTTGA
FGF7	GGGACCCAAGAGATGAAGAA	TTCACTTTCCACCCCTTTGA
α-SMA	TCTGTAAGGCCGGCTTTGC	TGTCCCATTCCCACCATCA
FSP1	AACTAAAGGAGCTGCTGACCC	TGTTGCTGTCCAAGTTGCTC
FAP	GCTGTGCTTGCCTTATTGGT	GTGTGAGTGCTCTCATTGTAT

### Soft-agarose assay

The colony formation assay was based on method described by Zheng et al. [45], with minor modifications. All assays were done in duplicate in 6-well plates (Costar). The bottom layer consisted 0.6% of L.M.P agarose (Invitrogen) in 2× DMEM supplemented with 10% FBS and was let to solidify. Next, 2-ml aliquots containing 0.3 ml 1.8 % agarose, 0.7 ml DMEM and 1 ml single cell suspension were layered on top of pre-coated wells, giving the final concentration of top agarose 0.3%. Depending on experiment, single cell suspension contained either 2.5×10^4^ fibroblasts / ml or 5×10^4^ SCC cells / ml. In order to determine the role of paracrine signaling between fibroblasts and carcinoma cells the assay was modified so that fibroblasts (2.5×10^4^ fibroblasts / ml) were first plated in wells as a monolayer and incubated for 24 hours. Medium was aspirated and bottom agarose was laid on top of semi-confluent cells and allowed to solidify, after which the top agarose with or without SCC cells (5×10^4^ SCC cells / ml) was overlaid. The plates were cultured at +37°C in 5%-CO_2_ incubator for 3 weeks without further feeding. The formed colonies were scored by calculating number of colonies in ten random views of 10× magnification in duplicate using an inverted microscope (Olympus CKX41) and photographed (Olympus DP12).

### SA-β-gal staining

Senescence-associated beta-galactosidase (SA-β−gal) activity was stained as described by Dimri et al. [46]. In brief, monolayer cultures of fibroblasts were fixed with 2% paraformaldehyde / 0.2% glutaraldehyde in PBS for 5 min, washed with PBS, and incubated with staining buffer (1 mg/ml X-gal, 150 mM NaCl, 2 mM MgCl_2_, 5 mM K_3_Fe(CN)_6_, 5 mM K_4_Fe(CN)_6_ in 40 mM phosphate buffer, pH 6) over night. Images of random views were captured at 4× magnification and the blue cells, indicating senescence, were calculated.

### Statistical analyses

All experiments were done in duplicates and repeated three times. The mean and SEM of all three experiments are shown. GraphPad Prism software was used to calculate statistical significance that was determined by unpaired Student's *t*-test.
